# Transcriptome profiling indicates varied gene responses to *Pasteurella multocida* mutant infections in cattle

**DOI:** 10.1371/journal.pone.0341813

**Published:** 2026-01-30

**Authors:** Hao Ma, Fred M. Tatum, Robert E. Briggs, Rohana P. Dassanayake, Tasia M. Kendrick, Eduardo Casas

**Affiliations:** 1 Ruminant Diseases and Immunology Research Unit, National Animal Disease Center, Agricultural Research Service, United States Department of Agriculture, Ames, Iowa, United States of America; 2 Department of Animal Science, College of Agriculture & Natural Resources, Michigan State University, East Lansing, Michigan, United States of America; Universitat fur Bodenkultur Wien, AUSTRIA

## Abstract

*Pasteurella multocida* is a pathogen that causes bovine respiratory disease, and the development of an effective vaccine is important for improving animal health. Live-attenuated vaccines induce a long-lasting immune response with minimal side effects. The objective of this study was to evaluate potential live vaccine candidates from three *P. multocida* mutants produced by separately disrupting the genes of filamentous hemagglutinin 2 (*fhaB2*)*,* hydrogenase-1 operon (*hyaE*)*,* and n-acylneuraminate-9-phosphatase (*nanP*) of a serogroup 3 strain (P1062, WT) by clinical testing and transcriptome analysis. Challenge with WT and the three mutants conferred protection against *P. multocida*, with less lung lesions (4.7–6.2%) compared to 22.4% in the sham group. Transcriptome analysis identified 807 differentially expressed protein-coding transcripts (DETs) in the blood and 6473 DETs in the liver compared to the sham, WT, and each of the mutants. In total, 15 and 64 differentially expressed microRNAs (DEmiRNAs) and 12 and 74 differentially expressed long non-coding RNAs (DElncRNAs) were identified in blood and liver, respectively. The DEmiRNAs were not significantly associated with the DETs within each comparison. DElncRNAs were associated with 12 and 170 DETs in blood and liver respectively. The greatest number of unique DETs were found between *hyaE* and sham groups in the liver, which agreed with the low colonization rate in the nares and palatine tonsils. For the DETs between sham and WT the under-enriched gene ontology terms in blood were all included in the liver for the DETs identified by WT vs. sham, *nanP* vs. sham, and *hyaE* vs. sham, and were related to the signaling pathway, stimulus, and sensory perceptions in biological processes with the molecular function of olfactory receptor activity. The number of identified DETs, decreased percentage of lung lesions, and colonization rates indicate that *fhaB2* could be a promising vaccine candidate.

## Introduction

Bovine respiratory disease (BRD) is a leading cause of morbidity and mortality in the dairy and beef industries, with an annual cost of approximately one billion dollars in the United States [1,2]. *Pasteurella multocida* is a bacterial pathogen that, in conjunction with multiple other factors such as viral infections and environmental and host factors, leads to compromised immune systems and triggers the development of BRD [3]. Strategies to control and prevent the onset of animal illness include decreasing stressors that contribute to the development of the disease, optimizing nutrition, increasing immunity, and administering antimicrobials [4]. The shortcomings of antimicrobial agents include their high cost, low efficacy, and the potential for microbes to develop antibiotic resistance [2,5]. The identification of a safe and effective vaccine candidate against *P. multocida* is critical for improving livestock health [6].

Transcriptome profiling has been extensively explored as one of the most important approaches to investigate the biological functions of diseases and pathogens. It has been used to uncover the protective mechanisms of vaccines by analysis of host immune responses [7–9]. Furthermore, many differentially expressed genes have previously been identified by RNA sequencing (RNA-seq) analysis as biomarkers for diagnostics and as a strategy to develop methods for their therapeutic targeting [10–13]. With recent advances in sequencing technology, in addition to protein coding genes, non-coding RNAs such as microRNAs (miRNAs) and long non-coding RNA (lncRNAs) have been identified as potential biomarkers and regulators at transcriptional and post-transcriptional levels [14,15]. MiRNAs are endogenous non-coding RNA molecules approximately 22 nucleotides in length. MiRNAs have been reported to negatively and positively regulate target gene expression [16–19]. As biomarkers, miRNAs are associated with various pathogenic infections [20–25]. LncRNAs are RNA polymerase II transcripts with a length greater than 200 nucleotides and low coding potential capacity. Extensive studies on the response of lncRNAs to diseases have been reported in several species, including porcine [26], bovine [27], rabbits [28], and chickens [29].

The interactions among genes, miRNAs, and LncRNAs were reported in many publications. Furthermore, studies on transcriptome changes in response to pathogenic infections have indicated that substantial temporal variations exist for different pathogens or isolated infections [30]. Shared and specific differentially expressed transcripts have been observed in different tissues of animals infected with the same pathogen [31,32]. Blood is important for maintaining homeostasis and is critical for immunity to defend the body against diseases. The liver is an essential metabolic organ that plays an important role in the innate immune response during infection by activating the cells residing in the liver [33,34]. The objective of the current study was to assess the clinical observations and differential expression of transcript variations in the blood and liver in response to *P. multocida* challenge in calves previously exposed to mutants with a potential virulence factor gene disrupted, to provide a foundation for potential vaccine candidate selection.

## Materials and methods

### Generation of *P. multocida* mutants

**Fig 1:**
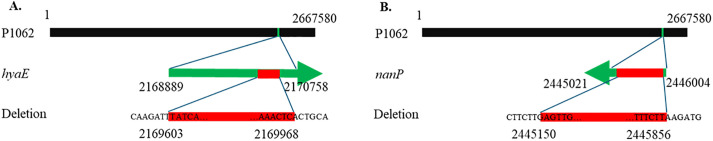
A schematic diagram of the relative location of the deletions to generate *hyaE* and *nan*P mutants. Panel A: *hyaE* deletion; Panel B: *nanP* deletion. The black lines represent the P1062 strain genome; the green lines represent the potential virulent factor genes; red represents the deleted fragment.

*Pasteurella multocida* strain P1062 (serotype A:3) was isolated from the pneumonic lung of a Holstein–Friesian calf that died from respiratory disease; its genome was sequenced in 2015 [35]. Three putative virulence factors were selected in the experiment, two of which, hydrogenase-1 operon protein (*hyaE*) and n-acylneuraminate-9-phosphatase (*nanP*), were partially deleted from the P1062 strain genome with 366 and 708 bp, respectively (Fig 1). Another one, filamentous hemagglutinin 2 gene (*fhaB2*) was described below.

DNA fragments containing the *hyaE* gene of P1062 were amplified with primer pairs (S1 Table) to produce an approximately 1,870-bp fragment containing the *hyaE* coding sequence. The polymerase chain reaction (PCR) product was cloned into the plasmid pCR2.1 (Invitrogen, La Jolla, CA, USA) and amplified in *Escherichia coli* Top10 (Invitrogen) cells, from which plasmid DNA was isolated using Spin-preps (Qiagen Inc. Valencia, CA). The *hyaE* sequence carried on pCR2.1 was subjected to PCR with deletion primers to produce a linear product with pCR2.1, flanked by upstream and downstream arms containing a deletion within *hyaE*. Linear DNA was treated with T4-DNA ligase, and the re-circularized plasmid was introduced into *E. coli* cells by electroporation and cultured for plasmid amplification. Following plasmid isolation, the purified plasmid was digested with *EcoRV* (sites contained in the PCR primers) and ligated to produce a deletion fragment, resulting in the removal of *hyaE* amino acids 239–359 and the substitution of isoleucine for leucine at the former 360 position. The *hyaE* fragment was transferred to a temperature-sensitive (Ts) plasmid derived from the endogenous plasmid, *M. haemolytica* pD70. The Ts plasmid and its application in producing gene replacements have been described in detail in a previous publication [36].

The *nanP* replacement arms used to generate the mutation in *P. multocida* P1062 were obtained by PCR using the primers listed in S1 Table. The primer sequences in red were hybridized with *P. multocida nanP* whereas those in black were included for cloning purposes. The PCR product was subjected to *BamH1* and *Sal I* digestion and inserted into the corresponding sites of the pBCSK plasmid (Stratgene, Inc., La Jolla, CA, USA). An in-frame deletion within *nanP* was introduced following digestion with *EcoRI,* which produced a 708 bp in-frame deletion within the *nanP* gene.

The P1062 gene *fhaB2* was inactivated using a temperature-sensitive (Ts) replacement plasmid and inserted with KanRTn903 element into a unique *Bgl II* site in the gene fragment. The detailed description of the P1062 insertion mutant following the same procedure as we did previously in P1059 [37]. All the mutants we generated were sequence verified.

### Experimental design

Animal care and sample collection were done according to the management protocol approved by the Institutional Animal Care and Use Committee of the National Animal Disease Center, in Ames, IA, United States (protocol no. ARS-23–1160).

Twenty, 8-wk-old commercial calves of 54–91 kg were housed in the Animal biological safety level 2 (ABSL2) facility at the National Animal Disease Center, Ames, IA, USA. The calves were randomly assigned to five groups, each with four animals. One animal in the control group (sham) was euthanized because of torsion that caused bloating. All calves were housed in 10 rooms under similar conditions, with two calves per room and measures in place to prevent the spread of pathogens between rooms and groups. The animals were fed a combination of Timothy hay cubes and a pelleted Growena ration (Purina Mills, Mills Summit, MO). Calves were allowed to acclimate to the space for approximately 1 wk prior to treatment application. At day zero, 10 ml of Earle’s Balanced Salt Solution (EBSS) containing 2.0–2.3 × 10^9^ colony forming units (cfu) *P. multocida* P1062 (WT) or each of the three mutants was delivered to nares and palatine tonsils (S1 Fig). The control group received only EBSS. On days 58 (half of the animals) and 59 (the other half), all animals were intratracheally administered 100 ml EBSS containing 2.2 × 10^9^ cfu of WT P1062 strain with a stable plasmid containing chloramphenicol acetyltransferase to the lungs, and the inoculum was then washed with an additional 100 ml of sterile EBSS. After 5 d of the WT challenge, the animals were euthanized by intravenous administration of sodium pentobarbital, and the liver samples were collected.

### Enzyme-linked immunosorbent assay and confirmation of infection

About 5 ml of jugular venous blood samples were collected weekly, and sera were tested for antibodies against *P. multocida* whole cells. *P. multocida* P1062 *hyaE* was grown to an optical density (OD)_600nm_ of 0.6 then diluted 1:10 with carbonate–bicarbonate buffer (C3041, Sigma-Aldrich, Inc. St. Louis, MO). A total of 100 µL of culture was used to coat the wells in 96-well plates. The plates were coated overnight at 4°C. The plates were washed once and blocked for 1 h with TBS-T20 (Sigma T9039) containing 0.5% fish gelatin and 1% horse serum. Serum was diluted 1:50 in TBS-T20 and loaded in quadruplicate (pools) or duplicate (individual). The plate was incubated for 2 h at 20–22°C and then washed three times with blocking buffer. Sheep anti-bovine IgA and IgM alkaline phosphatases (Sigma-Aldrich, Inc., St. Louis, MO, USA) were diluted 1:1000 in TBS-T20 buffer and incubated for 1 h. The plates were washed three times with blocking buffer. Alkaline phosphatase activity was detected using SigmaFast pNPP (N2770) (Sigma-Aldrich, Inc., St. Louis, MO, USA), and plates were read for 6–8 min at 405 nm using SpectraMax250 (Molecular Devices LLC., San Jose, CA). Serum samples from *P. multocida*-exposed calves diluted to 1:10, 1:40, 1:160, and 1:640 were used as standard positive controls for plate variation.

All of the calves were monitored daily for clinical signs of respiratory infection. Nasal bacterial shedding was assessed by taking a nasal swab, which was collected by inserting an individual 15-cm swab into each nostril until it was sufficiently saturated. Palatine tonsils were evaluated using an oral speculum and modified pipettes. Palatine tonsil washes were performed by flushing each crypt with 3 ml EBSS and immediately collecting the liquid. Samples were collected on arrival, on D0 (day of exposure), D3, and then weekly through to week 7. Nasal and tonsil swabs were directly plated (undiluted) on Columbia blood agar plates containing 5% defibrinated bovine blood. Additionally, nasal and tonsil samples were diluted ten-fold in EBSS, and 100 µL of each dilution was spread onto a blood agar plate (BAP). All plates were incubated overnight at 37 °C and growth was quantitated. The *hyaE* was the only mutation with an observable phenotype of less mass and slimier compared to the WT, *nanP*, and *fhaB2* because of the disruption of the capsule biosynthetic gene (S2 Fig) [37]. Confirmation of the strains was performed using PCR for each mutant and wild-type strain using the primers listed in S1 Table.

### Confirmation of lung infection and lesion evaluation

Infection in the lungs was confirmed using lung specimens diluted in a 1:10 weight:volume in EBSS. The specimens were homogenized using a Tekmar Tissumizer SDT1810 (TZ Supplies, Fleetwood, Pennsylvania, USA). Suspension and ten-fold dilutions were prepared in EBSS. The dilutions of 100 µL were spread in duplicate onto BAPs containing 5% defibrinated bovine blood with chloramphenicol and incubated overnight at 37 °C; strain growth was then quantified [38].

Lung lesion scores were estimated visually and by palpation in each lung lobe, including both consolidated areas and volumes that appeared grossly atelectatic. The total lung lesion scores were calculated as the sum of the eight individual lobe scores, where the estimated percentage of each lobe’s lung lesion volume was multiplied by an approximation of its contribution to air exchange as: right cranial lobe, 6%; cranial half of the right middle lobe, 5%; caudal half of the right middle lobe, 7%; right caudal lobe, 35%; accessory lobe, 4%; left cranial lobe, 4%; left middle lobe, 6%; and left caudal lobe, 32% [39].

### RNA isolation and sequencing

Total RNA was extracted from blood and liver samples collected at necropsy using the MagMAX^TM^ mirVana^TM^ Total RNA Isolation Kit (ThermoFisher Scientific, Waltham, MA, USA) and eluted in 100 μl of RNase-free water. The concentration and quality of small RNAs in each sample were determined using a 10–40 nucleotide gate on an Agilent 2100 Bioanalyzer Small RNA chip (Agilent Technologies, Santa Clara, CA, USA). Purified RNA extracted from each sample with RNA integrity number (RIN) of at least 7.5 was used to prepare individual libraries using the NEBNext Multiplex Small RNA Library Prep Kit (New England Biolabs, Ipswich, MA, USA). Library concentration and purification were performed using the QIAquick PCR Purification Kit (QIAGEN, Germantown, MD, USA). Each library between 135 and 170 bp was run on an Agilent 2100 Bioanalyzer High-Sensitivity DNA chip (Agilent, Santa Clara, CA, USA) to determine the quality and quantity of the prepared library. Then, 30 ng of each library was pooled, and the size was selected using AMPure XP beads (Beckman Coulter, Indianapolis, IN, USA). Following size selection, library pools were concentrated using a QIAquick PCR purification kit (QIAGEN, Germantown, MD, USA) and eluted in RNase-free water. An Agilent 2100 Bioanalyzer high-sensitivity DNA chip was used to determine the concentration of each library pool. The library pool was sequenced as single-end 50-bp reads using the Illumina HiSeq 3000 System (Illumina, San Diego, CA, USA).

For messenger RNA (mRNA) sequencing, individual libraries each with 1 µg and RIN > 7.5 were constructed using the NEB Next Ultra RNA Library Kit for Illumina and the NEBNext PolyA Magnetic Isolation Module (NEB, Ipswich, MA, USA). The library was enriched with 9 PCR cycles. After final clean-up with Agencourt AMPure XP Beads, 1 μl of the libraries were run on an Agilent 2000 bioanalyzer using the high sensitivity DNA chip to measure the quality and quantity of the libraries. Based on average size and concentration, individual libraries were pooled to an equal molar concentration (1.5 nm) and sequenced using an Illumina HiSeq 3000 sequencer at paired-end 2 × 100 bp at the Iowa State University DNA Sequencing Facility (Ames, IA, USA).

### Identification of differentially expressed miRNAs and transcripts

The bovine reference genome was downloaded from Ensembl Genes 102 (https://uswest.ensembl.org/info/data/ftp/index.html) and the miRNA precursor and mature sequences were downloaded from miRBase (http://www.mirbase.org, Release 22.1). FastQC (version 1.11.5) was used to evaluate blood and liver small non-coding RNA raw sequences [40], and Cutadapt (version 1.16 with Python 2.7.13) was used with Q30 to select high-quality sequences and remove adaptors and highly-presented junk sequences [41]. Known and novel miRNA sequences from blood and liver were quantified using miRDeep2 (version 0.1.3) as described previously [42,43]. The predicted novel miRNAs were presented in at least six samples among blood, liver, bronchial lymph node, palatine tonsil, and retropharyngeal lymph node tissues. The counted blood and liver miRNA reads were then used to identify differentially expressed miRNAs (DEmiRNAs) using the DESeq2 package in R (version 3.6.1) [44] with a false discovery rate of <0.05. Comparisons were conducted in the blood and liver between the treatments of *fhaB2* and WT, *hyaE* and WT, *nanP* and WT, sham and WT (or WT vs sham), *fhaB2* and sham, *hyaE* and sham, and *nanP* and sham. The small RNA sequences are available in the NCBI SRA under the BioProject accession number, PRJNA1241202.

Blood and liver sequencing reads were selected and evaluated in a manner similar to that of small non-coding RNA, as described above. Reads were mapped against the bovine reference genome using STAR aligner (version 2.7.2b) with the settings of outFilterMultimapNmax 30, alignSJoverhangMin 5, alignSJDBoverhangMin 3, outFilterScoreMinOverLread 0.50, and outFilterMatchNminOverLread 0.50 [45]. Raw transcript sequences were sorted using Samtools (version 1.9) [46] and then counted using RSEM (version 1.3.0) [47]. Raw counts were analyzed using the DESeq2 package [44] to identify differentially expressed protein-coding transcripts (DETs) with the criteria of Log_2_FC =>|1| and false discovery rate <0.05. Raw RNA-seq data were deposited in the NCBI SRA database under the BioProject accession number, PRJNA1241202.

### Target gene prediction

The bovine 3′ untranslated regions (UTR) were obtained by running the R package, “biomartr” (Version 0.9.0) [48]. Target genes of the differentially expressed miRNAs were separately predicted by miRanda (aug2010) and PITA [49,50]. The criteria to determine target transcripts are ΔG ≤ −15 kcal/mol for miRanda and energetic score ≤ −10 kcal/mol for PITA, where ΔG is the free energy of duplex formation from a completely dissociated state which was calculated using the Vienna package (version 1.0.125) [49]. The reported target genes were predicted using both programs.

Based on chromosomal coordinate information, the “*cis-*” interacted DETs of differentially expressed lncRNA (DElncRNA) were determined as those that were located adjacent to the DElncRNAs within 100-kb upstream and downstream on the same chromosome [27,35,51,52]. As three to four animals were used for each treatment, it was not appropriate to reliably predict *trans*-acting lncRNAs based on correlation coefficients.

### Gene functional analyses and statistical test

The transcripts listed in Ensembl Genes 102 were used as reference. Gene Ontology (GO) analysis of the transcripts of interest was performed using OmicsBox version 3.2.0 (www.biobam.com). Gene set enrichment was tested using Fisher’s exact test and Benjamini-Hochberg procedure was used for multiple testing corrections. The Fisher’s exact test for the number of DETs identified from each comparison, Wilcoxon signed-rank test for DEmiRNA and targeted gene, and Spearman correlation coefficients of the number of DElincRNAs and DETs were estimated using R (version 3.6.1) [53–55].

## Results

### Pathogen colonization, lung lesions, and immunoassay

*P. multocida* colonization test showed that the pathogens were detected in nares and palatine tonsil with a 50–100% rate for the animals inoculated with the WT and each of the mutants on day 3 post infection (Table 1). As expected, all animals in the sham group showed no *P. multocida* throughout the observation period. Pathogen localization was tested until day 42, which showed that the pathogens were identified in the nares from nasal swabs of at least half of the animals in the WT and *nanP* groups and 25% of the animals in the *fhaB2* group. In the palatine tonsils, *fhaB2* was detected in all animals from days 3–42; WT and *nanP* were identified in palatine tonsil washes in 0–100% and 25%−100% of the animals, respectively, whereas *hyaE* was undetectable after day 28 in the nares and after day 14 in the palatine tonsils.

**Table 1:** Percentage of animals with detected respective *Pasteurella multocida* WT or mutant strains in nares and palatine tonsils, post immunization.

Day	Nares					Palatine tonsils				
	Sham	*fhaB2*	*hyaE*	*nanP*	Wild type	Sham	*fhaB2*	*hyaE*	*nanP*	Wild type
**0**	0	0	0	0	0	0	0	0	0	0
**3**	0	50	50	75	75	0	100	75	100	100
**8**	0	25	50	50	75	0	100	100	100	100
**14**	0	75	25	50	100	0	100	75	100	100
**21**	0	25	0	50	50	0	100	0	75	100
**28**	0	50	25	50	75	0	100	0	100	75
**35**	0	25	0	50	75	0	100	0	75	0
**42**	0	25	0	50	50	0	100	0	25	75

**Fig 2:**
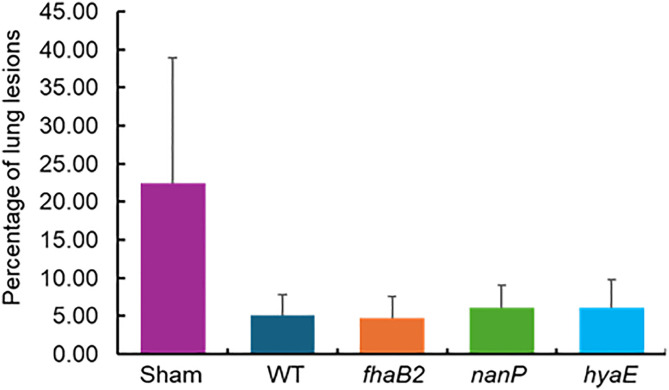
The percentage of lung lesions estimated for the animals treated with sham, wild type, *fhaB2*, *nanP2*, and *hyaE.* Error bar is the standard error of the mean.

All the animals, including the sham, were challenged intratracheally with *P. multocida* WT (chloramphenicol resistant). Within 48 hours of challenge, a pronounced clinical signs development was observed in the sham group compared to other groups such as fever, drooping ears, reduced feed intake and reduced response. The measurement of lung lesions after all animals were challenged with WT indicated that the protective effects of WT and the three mutants were substantially higher than in the sham group (Fig 2). The percentage of lung lesions was 22.43% in animals challenged with the sham and 4.75–6.16% in animals challenged with WT, *fhaB2*, *hyaE*, and *nanP* mutants.

**Fig 3:**
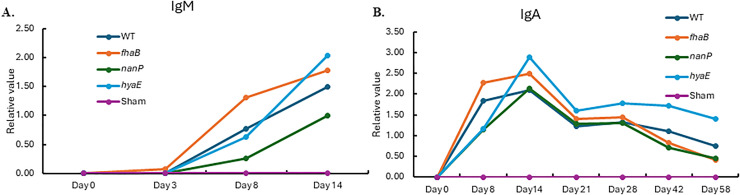
Immunoassay of serum with IgM and IgA for the animals challenged with sham, *fhaB2*, *nanP*, *hyaE*, and wild type. Panel A: IgM antibody; Panel B: IgA antibody.

Like colonization, IgM levels increased after day three in serum of the animals in the WT and mutant groups. IgA levels also increased after inoculation and reached a maximum on day 14, after which they decreased gradually (Fig 3).

### RNA sequencing

RNA sequencing of samples derived from the blood and liver of 19 animals generated an average of 61,337,572 raw reads, 61,326,291 cleaned reads, 52,943,364 uniquely mapped reads, and 3,424,118 multiple-mapped reads per sample (S2 Table). The average number of identified transcript was 18,984 from blood, and 21,756 from liver. Small-RNA sequencing produced an average of 16,854,619 raw reads, 16,696,252 clean reads, and 14,957,301 mapped reads per sample (S3 Table). The average numbers of identified mature and novel miRNA were 465 and 150 from blood, and 478 and 47 from liver respectively.

### Differentially expressed mRNAs, miRNAs, and lncRNAs

**Fig 4:**
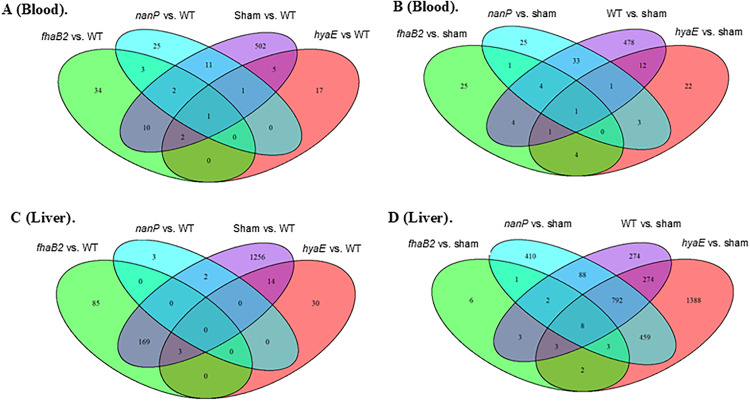
Venn diagram of differentially expressed transcripts in blood and liver. Comparison between each of the four treatments and wild type in blood **(A)** and liver **(C)**; Comparison between each of the four treatments and sham in blood **(B)** and liver **(D)**.

The raw reads were used to test the differences of the transcripts for each comparison between two groups of the animals. The analysis identified a greater number of DETs in the liver (6473) than in the blood (807) (Table 2 and S3 Fig). Similarly, 64 and 15 DEmiRNAs and 74 and 12 differentially expressed lncRNAs (DElncRNAs) were identified in the liver and blood samples, respectively. Shared and unique DETs are shown in Fig 4. The sham vs. WT contrast in the blood and liver showed an increased number of DETs compared to the mutant vs. WT. Fisher’s exact test showed extremely significant difference between the number of the DETs identified from sham vs. WT and each of the mutants vs. WT for both blood and liver (P-value < 2.2e^-16^). Blood and liver samples showed differences in the number of identified DETs, DElncRNAs, and DEmiRNAs.

**Table 2:** Number of differentially expressed transcript (DET), miRNA (DEmiRNA), and lncRNA (DELncRNA).

Tissue	Comparison	DET		DEmiRNA		DELncRN A	
		LFC > 1	LFC < −1	LFC > 0	LFC < 0	LFC > 1	LFC < −1
**Blood**	***fhaB2* vs. wild type**	19	33	0	2	1	1
	***hyaE* vs. wild type**	11	15	0	0	0	0
	***nanP* vs. wild type**	13	30	0	3	1	0
	**sham vs. wild type**	73	461	0	3	0	8
	***fhaB2* vs. sham**	23	17	1	2	0	0
	***hyaE* vs. sham**	22	22	1	1	0	0
	***nanP* vs. sham**	53	15	1	1	1	0
**Liver**	***fhaB2* vs. wild type**	43	214	5	6	0	4
	***hyaE* vs. wild type**	18	29	2	3	0	0
	***nanP* vs.wild type**	5	0	1	1	0	0
	**sham vs. wild type**	822	622	7	1	2	19
	***fhaB2* vs. sham**	11	17	0	1	1	0
	***hyaE* vs. sham**	1262	1667	9	16	27	6
	***nanP* vs. sham**	661	1102	5	7	11	4

LFC: Log fold change.

In liver, the number of DETs from *fhaB2* vs. WT was significantly smaller than that from sham vs. WT, but larger than that from *hyaE* vs. WT or *nanP* vs. WT (P-value <0.05), which indicated that *fhaB* induced relatively fewer changes in gene expression after the calves were infected by the WT and the mutants. The numbers of DETs from *hyaE* vs. WT and *nanP* vs. WT were smaller than those from *fhaB2* vs. WT, indicating that host responses to *hyaE* and *nanP* infections were closer to those of the WT. This result was consistent with the number of DETs from *fhaB2* vs. sham, which was significantly smaller compared to those from sham vs. WT, *hyaE* vs. sham, and *nanP* vs. sham (P-value < 2.2e^-16^). These comparisons confirmed that *fhaB2* caused less molecular variation than the WT, *hyaE*, and *nanP*. The number of DETs from all comparisons in blood showed a similar pattern as that in liver. In both blood and liver, a similar number of DETs were shared between *fhaB2* vs. sham, *hya*E vs. sham, and *nanP* vs. sham (Fig 4).

A similar pattern of DEmiRNAs and DElncRNA was observed in the blood and liver. A greater number of DElncRNAs were also identified in *hyaE* vs. sham [33], followed by sham vs. WT [21], and *nanP* vs. sham [15] in the liver. A larger number of DEmiRNAs [19] and DElncRNAs [71] were identified in the liver than in the blood (15 and 12, respectively). Of all comparisons in the liver, more DEmiRNAs and DElincRNAs were found in *hyaE* vs. sham, *nanP* vs. sham, *fhaB2* vs. WT, and sham vs. WT, with 25, 12, 11, and 8 DEmiRNAs, and 33, 15, 4, and 21 DElncRNAs, respectively.

### Functional analysis of DETs

Functional gene analysis of the DETs in blood resulted in 598, 147, 8, and 1 significantly enriched gene ontology terms (GOs) for sham vs. WT, *nanP* vs. sham, *fhaB2* vs. WT, and *fhaB2* vs. sham comparisons, respectively (Table 3). In the liver, 383, 212, 201, 21, and 3 GOs were significantly enriched for *hyaE* vs. sham, *nanP* vs. sham, WT vs. sham, *fhaB2* vs. WT, and *fhaB2* vs. sham, respectively. Although the comparisons showed a similar pattern of significantly enriched GOs in the blood and liver, excluding *hyaE* vs. sham, the over-enriched GO terms in the three GO categories varied greatly between the two tissues. Similar to the sham vs. WT comparison with over-enriched GOs under biological processes, only seven GOs were shared for a total of 458 in blood and 98 in the liver; for molecular function, only two were shared for a total of 41 in both blood and liver. In the cellular component category, only one was shared, with 28 in the blood and 23 in the liver (S4 Table).

**Table 3:** Total number of significantly enriched gene ontology terms in blood and liver.

Comparison	Blood	Liver
***fhaB2* vs. wild type**	8	21
***hyaE* vs. wild type**	0	0
***nanP* vs. wild type**	0	0
**sham vs. wild type**	598	201
***fhaB2* vs. sham**	1	3
***hyaE* vs. sham**	0	383
***nanP* vs. sham**	147	212

**Fig 5:**
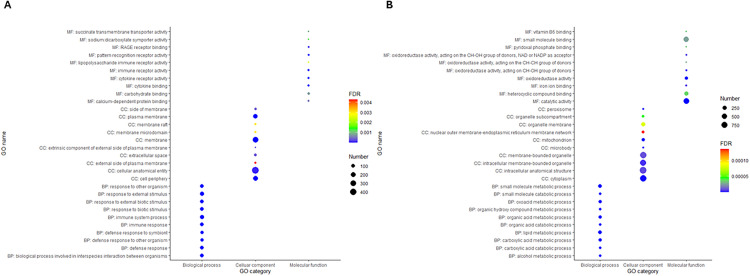
Top 10 significantly over enriched gene ontology terms associated with the DETs from sham vs. WT in blood and liver. Panel A: Blood; panel B: Liver. BP-Biological process; MF-Molecular function; CC-Cellular component.

In the blood, the most significantly over-enriched GOs associated with the DETs from sham vs. WT (based on adjusted p-values) were immune and defense responses and response to biotic stimulus, which were processed over membranes, cellular anatomical entities, and extracellular space (Fig 5 and S4 Table). The molecular functions of the enriched GOs were mainly involved in the activities of the immune system, cytokine receptor, pattern recognition receptor, succinate transmembrane transporter, sodium:dicarboxylate symporter, lipopolysaccharide immune receptor, binding of carbohydrates, cytokines, calcium-dependent proteins, and RAGE receptors. All the top enriched GOs were from the DETs, which were highly expressed in the sham compared to the wild-type group, which indicated that the downregulated DETs did not contribute to the enrichment of the top enriched GO terms. The top over-enriched GOs in all three categories were the same as those identified in the *nanP* vs. sham comparison (S4 Table).

In contrast to the over-enriched GO terms in blood, as described above, the liver showed considerably higher GO enrichment in the DETs identified from sham vs. WT, *hyaE* vs. sham, and *nanP* vs. sham, which were metabolic and catabolic processes of small molecules, lipids, oxoacids, organic acids, carboxylic acids, organic hydroxy compounds, and alcohols. The molecular functions of the DETs were catalytic and oxidoreductase activities, as well as small molecules, heterocyclic compounds, iron ions, pyridoxal phosphates, and vitamin B6 binding in the cytoplasm, mitochondria, organelle sub-compartments, peroxisomes, microbodies, intracellular anatomical structures, membrane-bound organelles, and organelle membranes. DETs contributed to the GO enrichment were highly expressed in WT, *nanP*, and *hyaE* animals compared to those in the sham group.

**Fig 6:**
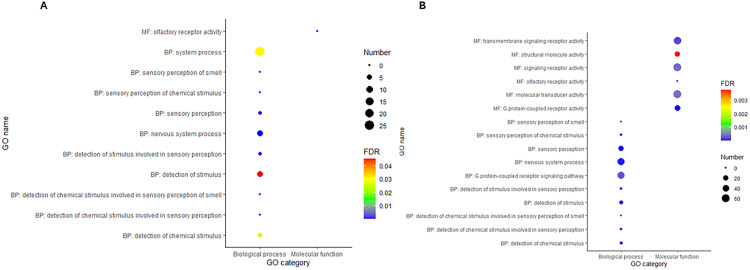
Top 10 significantly under enriched gene ontology terms associated with the DETs from sham vs. WT in blood and liver. Panel A: Blood; panel B: Liver. BP-Biological process; MF-Molecular function.

In contrast to the over-enriched GOs, the most under-enriched GOs of the DETs from sham vs. WT in the blood were also shared in the liver for sham vs. WT, *hyaE* vs. sham, and *nanP* vs. sham (Fig 6). These GOs include the detection of chemical stimuli, which are involved in sensory perception, nervous system processes, and the sensory perception of smell. The only under-enriched GO associated with molecular functions was olfactory receptor activity. For the shared GOs in blood and liver, the expression of the corresponding genes was suppressed in WT compared to sham in blood; however, in the liver, some transcripts associated with the GOs in the calves treated with sham, WT, *nanP,* and *hyaE* were suppressed, whereas other transcripts were highly expressed.

### The interaction of DEmiRNA, DElncRNA, and targeted DETs

To understand the possible associations between DEmiRNAs and DETs, the average percentages of target DETs within each comparison of DEmiRNAs were estimated (Table 4). The DEmiRNAs with Log_2_FC < 0 targeted more DETs within the comparisons than those out of the comparisons (Wilcoxon signed rank test, P-value = 0.0199); however, for DEmiRNAs with Log_2_FC > 0, there were no differences in the number of DETs targeted within each comparison and the DETs not in the comparison (P-value = 0.9102). The target difference for DEmiRNAs with Log_2_FC < 0 mainly arose from a higher percentage of targeted DETs with Log_2_FC < −1 (P-value = 0.0841) (Table 5). There were no obvious associations between DEmiRNAs and target DETs within each comparison in both the blood and liver because DEmiRNAs with Log_2_FC < 0 or >0 targeted DETs with both Log_2_FC < −1 and >1. These results indicate that DE miRNAs were associated with some DETs.

**Table 4:** Both PITA and MiRanda predicted percentages of target differentially expressed transcripts by the differentially expressed microRNAs.

Tissue	Comparison	All target DETs within comparison		All target DETs not in comparison	
		DEmiRNA with log_2_FC<0	DEmiRNA with log_2_FC>0	DEmiRNA with log_2_FC<0	DEmiRNA with log_2_FC>0
Blood	*fhaB2* vs. wild type	0.00		0.82	
	*hyaE* vs. wild type				
	*nanP* vs. wild type	4.65		2.81	
	sham vs. wild type	4.49		1.90	
	*fhaB2* vs. sham	1.25	0.00	2.80	0.31
	*hyaE* vs. sham	6.82	2.27	3.70	2.45
	*nanP* vs. sham	7.35	5.88	5.71	3.50
Liver	*fhaB2* vs. wild type	8.50	1.01	3.82	1.14
	*hyaE* vs. wild type	1.42	1.06	0.38	1.49
	*nanP* vs. wild type	0.00	0.00	0.81	0.18
	sham vs. wild type	1.04	0.51	0.88	0.45
	*fhaB2* vs. sham	8.82		4.29	
	*hyaE* vs. sham	1.31	2.74	0.96	2.00
	*nanP* vs. sham	0.57	3.02	0.49	2.66

Empty cell: No DEmiRNAs or DETs in the comparison.

**Table 5:** PITA and MiRanda predicted percentages of target differentially expressed transcripts by the differentially expressed microRNAs within each comparison.

Tissue	Comparison	Target DET with Log_2_FC < −1		Target DET with Log_2_FC > 1	
		DEmiRNA with log_2_FC<0	DEmiRNA with log_2_FC>0	DEmiRNA with log_2_FC<0	DEmiRNA with log_2_FC>0
Blood	*fhaB2* vs. wild type	0.00		0.00	
	*hyaE* vs. wild type				
	*nanP* vs. wild type	5.56		2.56	
	sham vs. wild type	4.63		3.65	
	*fhaB2* vs. sham	2.94	0.00	0.00	0.00
	*hyaE* vs. sham	4.55	4.55	9.09	0.00
	*nanP* vs. sham	13.33	13.33	5.66	3.77
Liver	*fhaB2* vs. wild type	9.58	1.21	3.10	0.00
	*hyaE* vs. wild type	0.00	1.72	3.70	0.00
	*nanP* vs. wild type			0.00	0.00
	sham vs. wild type	1.61	0.34	0.61	0.64
	*fhaB2* vs. sham	11.19		5.17	
	*hyaE* vs. sham	1.16	2.15	1.52	3.52
	*nanP* vs. sham	0.61	2.54	0.40	4.88

Empty cell means no DEmiRNAs or DETs.

The Spearman correlation coefficient estimated by the number of DElncRNAs and DETs in the blood and liver in all comparisons were highly significant (R_Spearman_ = 0.8694**). However, based on the localization of the lncRNAs and transcripts, the *cis*-regulation of the DElncRNAs on the DETs were different for the comparisons. In the blood, only sham vs. WT showed DElncRNAs that *cis*-regulated 12 of the DETs from the same comparison; in the liver, DElncRNAs from *fhaB2* vs. WT, sham vs. WT, *hyaE* vs. sham, and *nanP* vs. sham *cis*-regulated 170 of the DETs (S5 Table). Of all the DElncRNAs and DETs, only 37 DElncRNAs were localized to approximately 180 DETs within 100 kb. Based on the log_2_ FC values, 35 of the DElncRNAs and 167 DETs were in the same direction (positive–positive, or negative–negative), whereas 24 DElincRNAs and 32 DETs were in opposite directions.

## Discussion

The host–pathogen interactions clearly showed that there were dynamic processes from *P. multocida* invasion to dissemination. As the first line of defense against pathogens [56], IgA assay values increased immediately after *P. multocida* infection, reached a maximum on day 14, and then decreased gradually. However, the antibodies produced by the calves could recognize and neutralize the pathogen effectively until the challenge with the WT. Similar to clinical signs in which sham group showed the pronounced clinical responses, most lung lesions were observed in the animals in sham group. Approximately 22% lung lesions were in the sham group and about 4–6% lung lesions in the other four groups. Consistent with this, sham vs. WT in the blood and liver had an increased number of DETs compared to each mutant vs. the WT. This is a contrast between naïve animals and animals exposed to pathogens; therefore, the differences in gene expression are not surprising. Therefore, the animals were infected with each of the mutants, and the hosts developed resistance to the WT bacteria, causing the number of DETs from *nanP* vs. WT, *hyaE* vs. WT, and *fhaB2* vs. WT to be small.

Blood not only delivers necessary substances and removes waste, but it also performs coagulation, defense, and homeostasis maintenance [57,58]. The liver is important in the production of blood serum proteins, other critical proteins, and immune factors [33,34,59,60]. A larger number of DETs, DElncRNAs, and DEmiRNAs were identified in the liver than in blood, which may be related to the physiological functions of the two tissues [61]. The greatest number of DETs and significantly enriched GOs were identified in the liver by *hyaE* vs. sham (Tables 2 and 3), which was not only due to the specificity of the liver compared to blood, but was also caused by *hyaE* encoding the hyaluronic acid capsule, increasing adhesion and immune evasion of *P. multocida* [37]. The greatest number of unique DETs and enriched GOs from the hyaE vs. sham comparison was aligned with the difficulty of the mutant to colonize both the nares and palatine tonsils after 14 d of challenge (S1 Table and Fig 4D).

Recently, researchers have focused on identifying transcriptome changes in blood that might mirror changes in the liver; some genes involved in mitochondrial and immunological functions are co-regulated [61]. In a heat-stress study utilizing Sprague–Dawley rats, more DETs were identified in the liver than in the blood [62]. A similar result was reported in the present study by the genes suppressed in both tissues in WT with under-enriched GOs (Fig 5 and S4 Table); however, additional DETs in the liver were found to be significantly overexpressed in WT and shared the same biological processes and functions as the DETs in both blood and liver, which were suppressed in WT and overexpressed in sham. These results indicate the importance of the liver in response to pathogen infection.

There were more shared DETs in *fhaB2* vs. WT and sham vs. WT, which may indicate similar responses of calves to *fhaB2* and sham treatments (Fig 4C); however, the differences shown by the unique DETs between the two comparisons were still large. The unique DETs identified from the comparisons of *nanP* vs. sham, *hyaE* vs. sham, and WT vs. sham indicated that *nanP*, *hyaE*, and WT infections caused the animals to use different pathways to overexpress these genes. Nevertheless, the animals still had many common pathways, as reflected by the numerous shared DETs in the three comparisons (Fig 4). Based on shared and unique DETs from different comparisons, the responses to *nanP* and *hyaE* mutants were closer to the WT than *fhaB2*.

The miRNA and lncRNA have been known to regulate gene expression [14,15,63]. Regulation of gene expressions by miRNA and lncRNA has been widely reported [16–19,64–67]. PITA and miRanda were used for *in silico* prediction of target genes. For lncRNA targets, only *cis*-target genes have been reported as lncRNA *trans*-interacting genes were determined using correlation coefficients in many previous publications [27,68,69]. A limited number of calves were included in this study; therefore, a robust correlation was not guaranteed. These results support the association of DEmiRNAs and DElncRNA with DETs in both positive and negative directions. Similar to DElncRNAs, DEmiR-targeted DETs showed positive and negative relationships in different comparisons. With the dynamic changes in the transcriptome in response to pathogenic infections [9,25,30,31,70,71], future studies should focus on the profiling and interactions among mRNAs, miRNAs, and lncRNAs within different time frames and tissues after infection with each mutant and WT.

## Conclusion

Transcriptome analysis identified a greater number of differentially expressed transcripts, microRNAs, and long non-coding RNAs from the comparisons performed in the liver than in the blood for the calves inoculated with sham, WT, and the three generated mutants. The responses of calves infected with *nanP* and *hyaE* mutants were more similar to those of WT infected calves which supported by small number of DETs and not significantly enriched GOs identified by *nanP* vs. WT and *hyaE* vs. WT. In contrast, infection with *fhaB2* mutant resulted in fewer DETs from *fhaB2* vs. sham but more DETs from *fhaB2* vs. WT, indicating that *fhaB2* caused fewer transcriptomic changes than the other two mutants and elicited a response closer to sham animals. Together with the distinct transcriptomic profiles observed in blood and liver, as well as the reduced lung lesions and colonization, these findings suggest that *fhaB2* mutant could be a promising candidate vaccine for controlling *P. multocida* infections in cattle.

## Supporting information

S1 Fig Overview of timeline showing the interventions and sampling timepoints. (TIF)

S2 Fig Phenotypic difference of the *hyaE* compared to the WT, *nanP*, and *fhaB2.* The bacteria were grown on Trypticase™ Soy Agar (TSA II™) with 5% Sheep Blood (Fisher scientific, Waltham, Massachusetts, USA) overnight at 37°C.
(TIF)

S3 Fig Volcano plot of expressed genes with different comparisons in blood and liver. Panel A: Seven comparisons in blood; Panel B: Seven comparisons in liver.
(TIF)

S1 Table Primers used for *Pasteurella multocida* mutant generation and confirmation of colonization. (CSV)

S2 Table Messenger RNA sequencing statistics. (CSV)

S3 Table Small RNA sequencing statistics. (CSV)

S4 Table Significantly enriched gene ontology terms in blood and liver. (CSV)

S5 Table A list of differentially expressed LncRNA and closely localized differentially expressed transcript from each comparison. (CSV)

## References

[1] Kamel MS, Davidson JL, Verma MS. Strategies for Bovine Respiratory Disease (BRD) diagnosis and prognosis: a comprehensive overview. Animals (Basel). 2024;14(4):627. doi: 10.3390/ani14040627 38396598 PMC10885951

[2] Mostaan S, Ghasemzadeh A, Sardari S, Shokrgozar MA, Nikbakht Brujeni G, Abolhassani M, et al. Pasteurella multocida vaccine candidates: a systematic review. Avicenna J Med Biotechnol. 2020;12(3):140–7. 32695276 PMC7368114

[3] Dabo SM, Taylor JD, Confer AW. Pasteurella multocida and bovine respiratory disease. Anim Health Res Rev. 2007;8(2):129–50. doi: 10.1017/S1466252307001399 18218157

[4] Nickell JS, White BJ. Metaphylactic antimicrobial therapy for bovine respiratory disease in stocker and feedlot cattle. Vet Clin North Am Food Anim Pract. 2010;26(2):285–301. doi: 10.1016/j.cvfa.2010.04.006 20619185

[5] Andrés-Lasheras S, Jelinski M, Zaheer R, McAllister TA. Bovine respiratory disease: conventional to culture-independent approaches to studying antimicrobial resistance in North America. Antibiotics (Basel). 2022;11(4):487. doi: 10.3390/antibiotics11040487 35453238 PMC9025279

[6] Ran X, Meng X-Z, Geng H-L, Chang C, Chen X, Wen X, et al. Generation of porcine Pasteurella multocida ghost vaccine and examination of its immunogenicity against virulent challenge in mice. Microb Pathog. 2019;132:208–14. doi: 10.1016/j.micpath.2019.04.016 30980881

[7] Eshraghisamani R, Facciuolo A, De Buck J. Oral paratuberculosis vaccine efficacy and mucosal immunity in cattle. Vaccine. 2024;42(26):126447. doi: 10.1016/j.vaccine.2024.126447 39423453

[8] Artigas-Jerónimo S, Villar M, Estrada-Peña A, Alberdi P, de la Fuente J. Subolesin knockdown in tick cells provides insights into vaccine protective mechanisms. Vaccine. 2024;42(11):2801–9. doi: 10.1016/j.vaccine.2024.03.006 38508929

[9] He S, Liu S-Q, Teng X-Y, He J-Y, Liu Y, Gao J-H, et al. Comparative single-cell RNA sequencing analysis of immune response to inactivated vaccine and natural SARS-CoV-2 infection. J Med Virol. 2024;96(4):e29577. doi: 10.1002/jmv.29577 38572977

[10] Cao H, Fang C, Wang Q, Liu L-L, Liu W-J. Transcript characteristics on the susceptibility difference of bovine respiratory disease. Int J Genomics. 2023;2023:9934684. doi: 10.1155/2023/9934684 37180342 PMC10175020

[11] Casamassimi A, Federico A, Rienzo M, Esposito S, Ciccodicola A. Transcriptome profiling in human diseases: new advances and perspectives. Int J Mol Sci. 2017;18(8). 10.3390/ijms18081652 PMC5578042 28758927

[12] Porcu E, Sadler MC, Lepik K, Auwerx C, Wood AR, Weihs A, et al. Differentially expressed genes reflect disease-induced rather than disease-causing changes in the transcriptome. Nat Commun. 2021;12(1):5647. doi: 10.1038/s41467-021-25805-y 34561431 PMC8463674

[13] Horlock AD, Piersanti RL, Ramirez-Hernandez R, Yu F, Ma Z, Jeong KC, et al. Uterine infection alters the transcriptome of the bovine reproductive tract three months later. Reproduction. 2020;160(1):93–107. doi: 10.1530/REP-19-0564 32422601 PMC7291824

[14] Statello L, Guo C-J, Chen L-L, Huarte M. Gene regulation by long non-coding RNAs and its biological functions. Nat Rev Mol Cell Biol. 2021;22(2):96–118. doi: 10.1038/s41580-020-00315-9 33353982 PMC7754182

[15] Bartel DP. MicroRNAs: genomics, biogenesis, mechanism, and function. Cell. 2004;116(2):281–97. doi: 10.1016/s0092-8674(04)00045-5 14744438

[16] Skaftnesmo KO, Edvardsen RB, Furmanek T, Crespo D, Andersson E, Kleppe L, et al. Integrative testis transcriptome analysis reveals differentially expressed miRNAs and their mRNA targets during early puberty in Atlantic salmon. BMC Genomics. 2017;18(1):801. doi: 10.1186/s12864-017-4205-5 29047327 PMC5648517

[17] Ma F, Liu X, Li D, Wang P, Li N, Lu L, et al. MicroRNA-466l upregulates IL-10 expression in TLR-triggered macrophages by antagonizing RNA-binding protein tristetraprolin-mediated IL-10 mRNA degradation. J Immunol. 2010;184(11):6053–9. doi: 10.4049/jimmunol.0902308 20410487

[18] Place RF, Li L-C, Pookot D, Noonan EJ, Dahiya R. MicroRNA-373 induces expression of genes with complementary promoter sequences. Proc Natl Acad Sci U S A. 2008;105(5):1608–13. doi: 10.1073/pnas.0707594105 18227514 PMC2234192

[19] Giraldez AJ, Mishima Y, Rihel J, Grocock RJ, Van Dongen S, Inoue K, et al. Zebrafish MiR-430 promotes deadenylation and clearance of maternal mRNAs. Science. 2006;312(5770):75–9. doi: 10.1126/science.1122689 16484454

[20] Han SA, Jhun BW, Kim SY, Moon SM, Yang B, Kwon OJ. miRNA expression profiles and potential as biomarkers in nontuberculous mycobacterial pulmonary disease. Sci Rep. 2020;10(1). 10.1038/s41598-020-60132-0 PMC7035291 32081976

[21] Anadol E, Schierwagen R, Elfimova N, Tack K, Schwarze-Zander C, Eischeid H, et al. Circulating microRNAs as a marker for liver injury in human immunodeficiency virus patients. Hepatology. 2015;61(1):46–55. doi: 10.1002/hep.27369 25125218

[22] Baluni M, Ghildiyal S, Singh D, Himanshu Reddy D, Kumar R, Dhole TN. Increased serum microRNA-29b expression and bad recovery in Japanese encephalitis virus infected patients; A new component to improve the disease recovery. J Neuroimmunol. 2018;323:56–61. doi: 10.1016/j.jneuroim.2018.07.014 30196835

[23] Bellingham SA, Coleman BM, Hill AF. Small RNA deep sequencing reveals a distinct miRNA signature released in exosomes from prion-infected neuronal cells. Nucleic Acids Res. 2012;40(21):10937–49. doi: 10.1093/nar/gks832 22965126 PMC3505968

[24] Farr RJ, Rootes CL, Rowntree LC, Nguyen THO, Hensen L, Kedzierski L, et al. Altered microRNA expression in COVID-19 patients enables identification of SARS-CoV-2 infection. PLoS Pathog. 2021;17(7):e1009759. doi: 10.1371/journal.ppat.1009759 34320031 PMC8318295

[25] Andreassen R, Woldemariam NT, Egeland IØ, Agafonov O, Sindre H, Høyheim B. Identification of differentially expressed Atlantic salmon miRNAs responding to salmonid alphavirus (SAV) infection. BMC Genomics. 2017;18(1):349. doi: 10.1186/s12864-017-3741-3 28472924 PMC5418855

[26] Fang M, Yang Y, Wang N, Wang A, He Y, Wang J, et al. Genome-wide analysis of long non-coding RNA expression profile in porcine circovirus 2-infected intestinal porcine epithelial cell line by RNA sequencing. PeerJ. 2019;7:e6577. doi: 10.7717/peerj.6577 30863688 PMC6408913

[27] Gao X, Niu C, Wang Z, Jia S, Han M, Ma Y, et al. Comprehensive analysis of lncRNA expression profiles in cytopathic biotype BVDV-infected MDBK cells provides an insight into biological contexts of host-BVDV interactions. Virulence. 2021;12(1):20–34. doi: 10.1080/21505594.2020.1857572 33258421 PMC7781660

[28] Hu JQ, Li WQ, Huang B, Zhao QY, Fan XZ. The profiles of long non-coding RNA and mRNA transcriptome reveals the genes and pathway potentially involved in infection of New Zealand rabbits. Front Vet Sci. 2021;8. 10.3389/fvets.2021.591273 PMC8131872 34026883

[29] Yin L, Shen XH, Yin DD, Hou HY, Wang JR, Zhao RH. Integrated analysis of noncoding RNAs and mRNAs reveals their potential roles in chicken spleen response to infection. Res Vet Sci. 2023;164. 10.1016/j.rvsc.2023.105029 37769515

[30] Salcedo-Porras N, Oliveira PL, Guarneri AA, Lowenberger C. A fat body transcriptome analysis of the immune responses of Rhodnius prolixus to artificial infections with bacteria. Parasit Vectors. 2022;15(1):269. doi: 10.1186/s13071-022-05358-9 35906633 PMC9335980

[31] Oh S-I, Sheet S, Bui VN, Dao DT, Bui NA, Kim T-H, et al. Transcriptome profiles of organ tissues from pigs experimentally infected with African swine fever virus in early phase of infection. Emerg Microbes Infect. 2024;13(1):2366406. doi: 10.1080/22221751.2024.2366406 38847223 PMC11210422

[32] Li H, Zhao J, Li Y, Dong Z, Lin S, Guo B, et al. Transcriptome analysis reveals tissue-specific responses of Mytilus unguiculatus to Vibrio alginolyticus infection. Fish Shellfish Immunol. 2024;144:109301. doi: 10.1016/j.fsi.2023.109301 38110106

[33] Heymann F, Tacke F. Immunology in the liver--from homeostasis to disease. Nat Rev Gastroenterol Hepatol. 2016;13(2):88–110. doi: 10.1038/nrgastro.2015.200 26758786

[34] Ackermann MR, Derscheid R, Roth JA. Innate immunology of bovine respiratory disease. Vet Clin North Am Food Anim Pract. 2010;26(2):215–28. doi: 10.1016/j.cvfa.2010.03.001 20619180 PMC2904317

[35] Abrahante JE, Hunter SS, Maheswaran SK, Hauglund MJ, Tatum FM, Briggs RE. Draft genome sequence of Pasteurella multocida isolate P1062, isolated from bovine respiratory disease. Genome Announc. 2015;3(5):e01254-15. doi: 10.1128/genomeA.01254-15 26494687 PMC4616194

[36] Tatum FM, Yersin AG, Briggs RE. Construction and virulence of a Pasteurella multocida fhaB2 mutant in turkeys. Microb Pathog. 2005;39(1–2):9–17. doi: 10.1016/j.micpath.2005.05.003 15998577

[37] Gao P, Wang L, Wang S, Li G, Yi C, Wang Y, et al. The activity of hyaD contributed to the virulence of avian Pasteurella multocida. Microb Pathog. 2024;193:106768. doi: 10.1016/j.micpath.2024.106768 38960217

[38] Menghwar H, Tatum FM, Briggs RE, Goldkamp AK, Chriswell BO, Kanipe C, et al. Mannheimia haemolytica isogenic capsular and LPS-sialylation gene deletion mutants are attenuated in a calf lung challenge model. Microbiol Spectr. 2025;13(6):e0028325. doi: 10.1128/spectrum.00283-25 40265950 PMC12131826

[39] Briggs RE, Billing SR, Boatwright WD Jr, Chriswell BO, Casas E, Dassanayake RP, et al. Protection against Mycoplasma bovis infection in calves following intranasal vaccination with modified-live Mannheimia haemolytica expressing Mycoplasma antigens. Microb Pathog. 2021;161(Pt A):105159. doi: 10.1016/j.micpath.2021.105159 34454023

[40] Wingett SW, Andrews S. FastQ Screen: a tool for multi-genome mapping and quality control. F1000Res. 2018;7:1338. doi: 10.12688/f1000research.15931.2 30254741 PMC6124377

[41] Martin M. Cutadapt removes adapter sequences from high-throughput sequencing reads. EMBnetjournal. 2011;17(3).

[42] Friedländer MR, Mackowiak SD, Li N, Chen W, Rajewsky N. miRDeep2 accurately identifies known and hundreds of novel microRNA genes in seven animal clades. Nucleic Acids Res. 2012;40(1):37–52. doi: 10.1093/nar/gkr688 21911355 PMC3245920

[43] Putz EJ, Putz AM, Jeon H, Lippolis JD, Ma H, Reinhardt TA, et al. MicroRNA profiles of dry secretions through the first three weeks of the dry period from Holstein cows. Sci Rep. 2019;9(1):19658. doi: 10.1038/s41598-019-56193-5 31873189 PMC6928067

[44] Love MI, Huber W, Anders S. Moderated estimation of fold change and dispersion for RNA-seq data with DESeq2. Genome Biol. 2014;15(12):550. doi: 10.1186/s13059-014-0550-8 25516281 PMC4302049

[45] Dobin A, Davis CA, Schlesinger F, Drenkow J, Zaleski C, Jha S, et al. STAR: ultrafast universal RNA-seq aligner. Bioinformatics. 2013;29(1):15–21. doi: 10.1093/bioinformatics/bts635 23104886 PMC3530905

[46] Li H, Handsaker B, Wysoker A, Fennell T, Ruan J, Homer N, et al. The sequence alignment/map format and SAMtools. Bioinformatics. 2009;25(16):2078–9. doi: 10.1093/bioinformatics/btp352 19505943 PMC2723002

[47] Li B, Dewey CN. RSEM: accurate transcript quantification from RNA-Seq data with or without a reference genome. BMC Bioinform. 2011;12:323. doi: 10.1186/1471-2105-12-323 21816040 PMC3163565

[48] Drost HG, Paszkowski J. Biomartr: genomic data retrieval with R. Bioinformatics. 2017;33(8):1216–7. 28110292 10.1093/bioinformatics/btw821 PMC5408848

[49] Enright AJ, John B, Gaul U, Tuschl T, Sander C, Marks DS. MicroRNA targets in Drosophila. Genome Biol. 2003;5(1):R1. doi: 10.1186/gb-2003-5-1-r1 14709173 PMC395733

[50] Kertesz M, Iovino N, Unnerstall U, Gaul U, Segal E. The role of site accessibility in microRNA target recognition. Nat Genet. 2007;39(10):1278–84. doi: 10.1038/ng2135 17893677

[51] Ma Q, Li L, Tang Y, Fu Q, Liu S, Hu S, et al. Analyses of long non-coding RNAs and mRNA profiling through RNA sequencing of MDBK cells at different stages of bovine viral diarrhea virus infection. Res Vet Sci. 2017;115:508–16. doi: 10.1016/j.rvsc.2017.09.020 28968572

[52] Jiao H, Shuai X, Luo Y, Zhou Z, Zhao Y, Li B, et al. Deep insight into long non-coding RNA and mRNA transcriptome profiling in HepG2 cells expressing genotype IV swine hepatitis E virus ORF3. Front Vet Sci. 2021;8:625609. doi: 10.3389/fvets.2021.625609 33996960 PMC8116512

[53] Zhang L-N, Chen J-Y, Liu Y-X, Zhang Y, Hong L-L, Li X-X, et al. Identification of lncRNA dual targeting PD-L1 and PD-L2 as a novel prognostic predictor for gastric cancer. Front Oncol. 2024;14:1341056. doi: 10.3389/fonc.2024.1341056 39525623 PMC11544118

[54] Kolacinska A, Morawiec J, Fendler W, Malachowska B, Morawiec Z, Szemraj J, et al. Association of microRNAs and pathologic response to preoperative chemotherapy in triple negative breast cancer: preliminary report. Mol Biol Rep. 2014;41(5):2851–7. doi: 10.1007/s11033-014-3140-7 24469723 PMC4013446

[55] Li M, Yang L, Wang Y, Zhang L. Comprehensive analysis of diagnostic biomarkers related to histone acetylation in acute myocardial infarction. BMC Med Genomics. 2025;18(1):75. doi: 10.1186/s12920-025-02135-2 40251588 PMC12335776

[56] Nihei Y, Suzuki H, Suzuki Y. Current understanding of IgA antibodies in the pathogenesis of IgA nephropathy. Front Immunol. 2023;14:1165394. doi: 10.3389/fimmu.2023.1165394 37114051 PMC10126238

[57] Nicolai L, Gaertner F, Massberg S. Platelets in host defense: experimental and clinical insights. Trends Immunol. 2019;40(10):922–38. doi: 10.1016/j.it.2019.08.004 31601520

[58] Pan K, Zhu Y, Chen P, Yang K, Chen Y, Wang Y, et al. Biological functions and biomedical applications of extracellular vesicles derived from blood cells. Free Radic Biol Med. 2024;222:43–61. doi: 10.1016/j.freeradbiomed.2024.06.002 38848784

[59] Abbasi B, Hayat A, Lyons M, Gupta A, Gupta S. Serum protein and electrolyte imbalances are associated with chemotherapy induced neutropenia. Heliyon. 2022;8(7):e09949. doi: 10.1016/j.heliyon.2022.e09949 35865973 PMC9293742

[60] Robinson MW, Harmon C, O’Farrelly C. Liver immunology and its role in inflammation and homeostasis. Cell Mol Immunol. 2016;13(3):267–76. doi: 10.1038/cmi.2016.3 27063467 PMC4856809

[61] Zhang L, Bushel PR, Chou J, Zhou T, Watkins PB. Identification of identical transcript changes in liver and whole blood during acetaminophen toxicity. Front Genet. 2012;3:162. doi: 10.3389/fgene.2012.00162 22973295 PMC3432993

[62] Dou J, Sammad A, Cánovas A, Schenkel F, Usman T, Muniz MMM, et al. Identification of novel mRNA isoforms associated with acute heat stress response using RNA sequencing data in Sprague Dawley rats. Biology (Basel). 2022;11(12):1740. doi: 10.3390/biology11121740 36552250 PMC9774719

[63] Sebastian-delaCruz M, Gonzalez-Moro I, Olazagoitia-Garmendia A, Castellanos-Rubio A, Santin I. The role of lncRNAs in gene expression regulation through mRNA stabilization. Noncoding RNA. 2021;7(1):3. doi: 10.3390/ncrna7010003 33466464 PMC7839045

[64] Ma J, Zhang P, Wang Y, Lu M, Cao K, Wei S, et al. LncRNA HAR1A inhibits non-small cell lung cancer growth by downregulating c-MYC transcripts and facilitating its proteasomal degradation. Int Immunopharmacol. 2024;142(Pt B):113264. doi: 10.1016/j.intimp.2024.113264 39340992

[65] Vanamamalai VK, Priyanka E, Kannaki TR, Sharma S. Integrative study of chicken lung transcriptome to understand the host immune response during Newcastle disease virus challenge. Front Cell Infect Microbiol. 2024;14:1368887. doi: 10.3389/fcimb.2024.1368887 39290979 PMC11405381

[66] Haidar M, Tajeri S, Momeux L, Mourier T, Ben-Rached F, Mfarrej S, et al. miR-34c-3p Regulates protein kinase A activity independent of cAMP by dicing prkar2b transcripts in Theileria annulata-infected leukocytes. mSphere. 2023;8(2):e0052622. doi: 10.1128/msphere.00526-22 36847534 PMC10117149

[67] Hilker RE, Pan B, Zhan X, Li J. MicroRNA-21 enhances estradiol production by inhibiting WT1 expression in granulosa cells. J Mol Endocrinol. 2021;68(1):11–22. doi: 10.1530/JME-21-0162 34665763

[68] Fang M, Yang Y, Wang N, Wang A, He Y, Wang J, et al. Genome-wide analysis of long non-coding RNA expression profile in porcine circovirus 2-infected intestinal porcine epithelial cell line by RNA sequencing. PeerJ. 2019;7:e6577. doi: 10.7717/peerj.6577 30863688 PMC6408913

[69] Liu P, Zhang Y, Zou C, Yang C, Pan G, Ma L, et al. Integrated analysis of long non-coding RNAs and mRNAs reveals the regulatory network of maize seedling root responding to salt stress. BMC Genomics. 2022;23(1):50. doi: 10.1186/s12864-021-08286-7 35026983 PMC8756644

[70] Much C, Lasda EL, Pereira IT, Vallery TK, Ramirez D, Lewandowski JP, et al. The temporal dynamics of lncRNA Firre-mediated epigenetic and transcriptional regulation. Nat Commun. 2024;15(1):6821. doi: 10.1038/s41467-024-50402-0 39122712 PMC11316132

[71] Sarropoulos I, Marin R, Cardoso-Moreira M, Kaessmann H. Developmental dynamics of lncRNAs across mammalian organs and species. Nature. 2019;571(7766):510–4. doi: 10.1038/s41586-019-1341-x 31243368 PMC6660317

