# Supplementary figures and images for: Transcriptome profiling indicates varied gene responses to Pasteurella multocida mutant infections in cattle
Source: PLoS One. 2026 Jan 30;21(1):e0341813. doi: 10.1371/journal.pone.0341813 (PMC12857936; doi:10.1371/journal.pone.0341813)

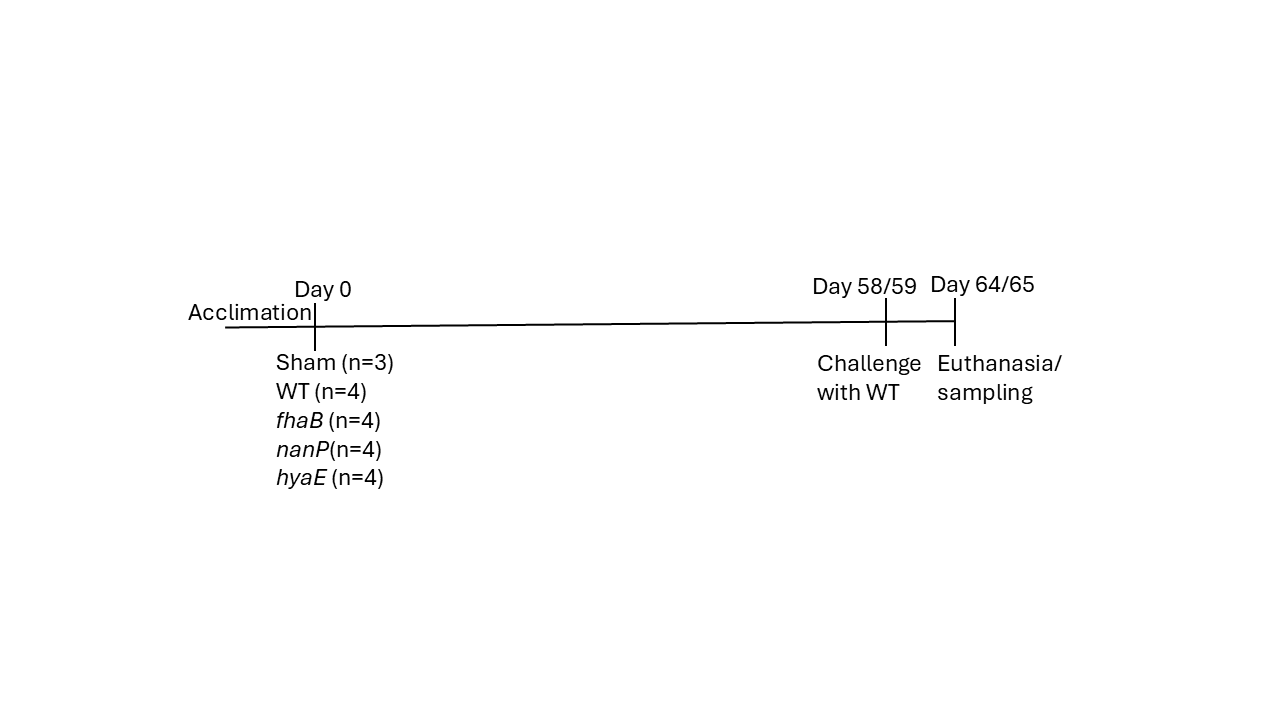

Supplement: S1 Fig — (TIF) [file pone.0341813.s001.tif]

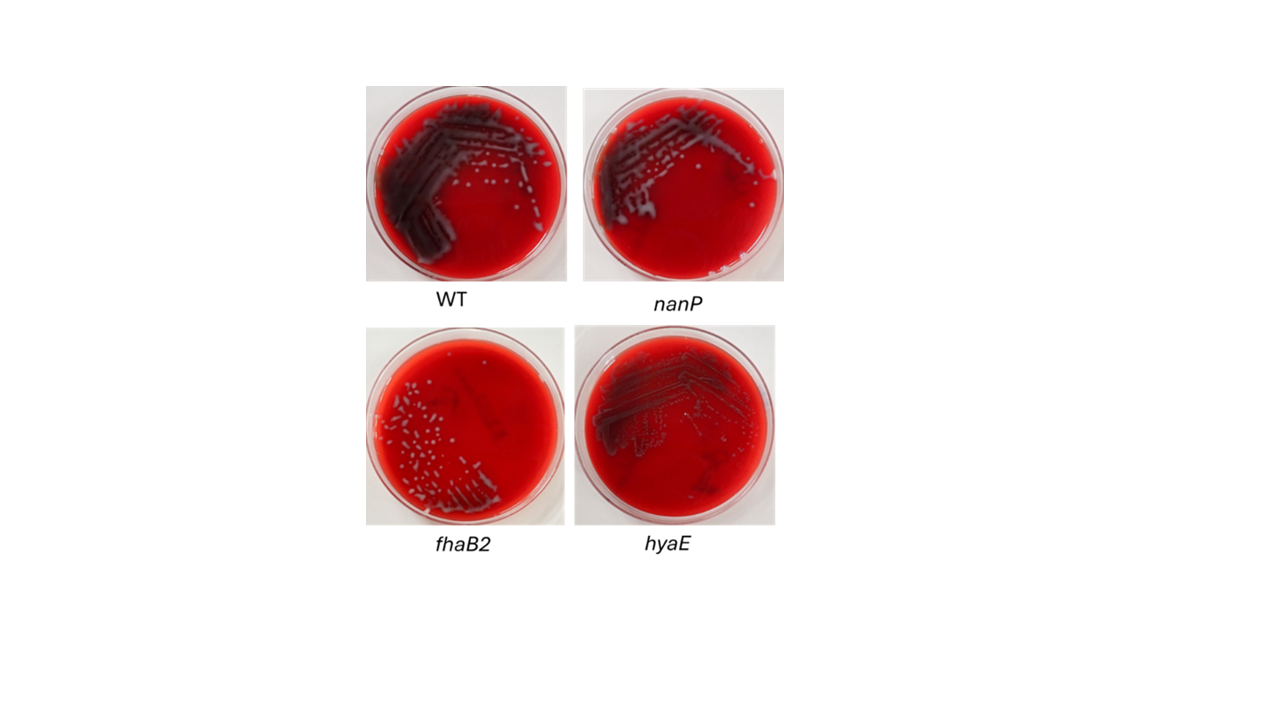

Supplement: S2 Fig — The bacteria were grown on Trypticase™ Soy Agar (TSA II™) with 5% Sheep Blood (Fisher scientific, Waltham, Massachusetts, USA) overnight at 37°C. (TIF) [file pone.0341813.s002.tif]

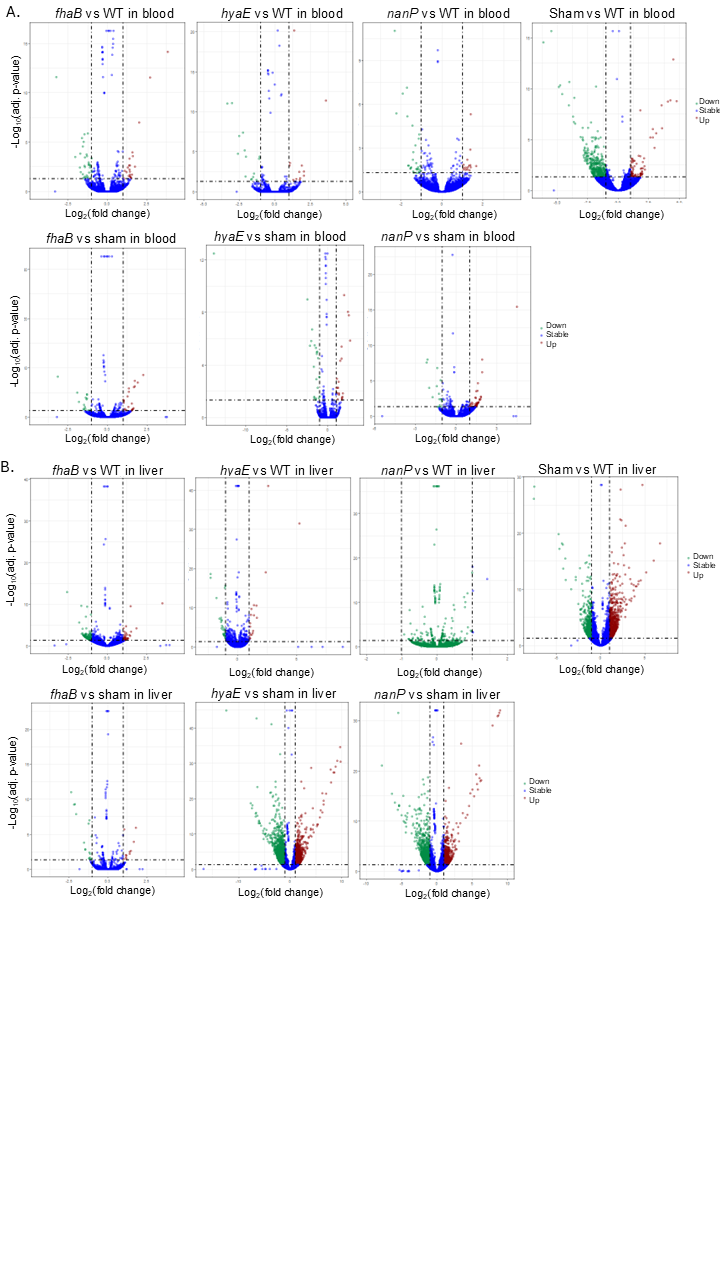

Supplement: S3 Fig — Panel A: Seven comparisons in blood; Panel B: Seven comparisons in liver. (TIF) [file pone.0341813.s003.tif]
